# The Mirasol Evaluation of Reduction in Infections Trial (MERIT): study protocol for a randomized controlled clinical trial

**DOI:** 10.1186/s13063-022-06137-8

**Published:** 2022-04-04

**Authors:** Ronnie Kasirye, Heather A. Hume, Evan M. Bloch, Irene Lubega, Dorothy Kyeyune, Ruchee Shrestha, Henry Ddungu, Hellen Wambongo Musana, Aggrey Dhabangi, Joseph Ouma, Priscilla Eroju, Telsa de Lange, Michael Tartakovsky, Jodie L. White, Ceasar Kakura, Mary Glenn Fowler, Philippa Musoke, Monica Nolan, M. Kate Grabowski, Lawrence H. Moulton, Susan L. Stramer, Denise Whitby, Peter A. Zimmerman, Deo Wabwire, Isaac Kajja, Jeffrey McCullough, Raymond Goodrich, Thomas C. Quinn, Robert Cortes, Paul M. Ness, Aaron A. R. Tobian

**Affiliations:** 1 MUJHU Research Collaboration, Kampala, Uganda; 2 Department of Pediatrics, University of Montreal, Montréal, QC Canada; 3 Department of Pathology, School of Medicine, Johns Hopkins University, Baltimore, MD USA; 4 Uganda Blood Transfusion Services, Kampala, Uganda; 5 Uganda Cancer Institute, Kampala, Uganda; 6 Child Health and Development Centre, Makerere University College of Health Sciences, Kampala, Uganda; 7 National Institute of Allergy and Infectious Diseases Office of Cyber Infrastructure and Computational Biology, Bethesda, MD USA; 8 Makerere University, Kampala, Uganda; 9 Department of International Health, School of Public Health, Johns Hopkins University, Baltimore, MD USA; 10 Department of Scientific Affairs, American Red Cross, Gaithersburg, MD USA; 11 Leidos Biomedical Research, AIDS and Cancer Virus Program, Frederick National Laboratory for Cancer Research, Frederick, MD USA; 12 The Center for Global Health & Diseases, Pathology Department, Case Western Reserve University, Cleveland, OH USA; 13 Department of Orthopaedics, Makerere University College of Health Sciences, Kampala, Uganda; 14 College of Health Solutions, Arizona State University, Phoenix, AZ USA; 15 Department of Microbiology, Immunology and Pathology, Colorado State University, Fort Collins, CO USA; 16 Division of Intramural Research, National Institute of Allergy and Infectious Diseases, National Institutes of Health, Bethesda, MD USA; 17 Terumo BCT, Lakewood, CO USA; 18 Department of Paediatrics and Child Health, College of Health Sciences, Makerere University, Kampala, Uganda

**Keywords:** Mirasol, Pathogen reduction, Transfusion-transmitted infections, Randomized controlled trial, Uganda, Sub-Saharan Africa

## Abstract

**Background:**

Transfusion-transmitted infections (TTIs) are a global health challenge. One new approach to reduce TTIs is the use of pathogen reduction technology (PRT). In vitro, Mirasol PRT reduces the infectious load in whole blood (WB) by at least 99%. However, there are limited in vivo data on the safety and efficacy of Mirasol PRT. The objective of the Mirasol Evaluation of Reduction in Infections Trial (MERIT) is to investigate whether Mirasol PRT of WB can prevent seven targeted TTIs (malaria, bacteria, human immunodeficiency virus, hepatitis B virus, hepatitis C virus, hepatitis E virus, and human herpesvirus 8).

**Methods:**

MERIT is a randomized, double-blinded, controlled clinical trial. Recruitment started in November 2019 and is expected to end in 2024. Consenting participants who require transfusion as medically indicated at three hospitals in Kampala, Uganda, will be randomized to receive either Mirasol-treated WB (*n* = 1000) or standard WB (*n* = 1000). TTI testing will be performed on donor units and recipients (pre-transfusion and day 2, day 7, week 4, and week 10 after transfusion). The primary endpoint is the cumulative incidence of one or more targeted TTIs from the Mirasol-treated WB vs. standard WB in a previously negative recipient for the specific TTI that is also detected in the donor unit. Log-binomial regression models will be used to estimate the relative risk reduction of a TTI by 10 weeks associated with Mirasol PRT. The clinical effectiveness of Mirasol WB compared to standard WB products in recipients will also be evaluated.

**Discussion:**

Screening infrastructure for TTIs in low-resource settings has gaps, even for major TTIs. PRT presents a fast, potentially cost-effective, and easy-to-use technology to improve blood safety. MERIT is the largest clinical trial designed to evaluate the use of Mirasol PRT for WB. In addition, this trial will provide data on TTIs in Uganda.

**Trial registration:**

Mirasol Evaluation of Reduction in Infections Trial (MERIT) NCT03737669. Registered on 9 November 2018

## Introduction

Transfusion-transmitted infections (TTIs) of known and unknown pathogens are a global health challenge [1]. The United Nations Programme on HIV and AIDS (UNAIDS) has suggested up to 1% of new HIV infections per year may be attributable to transfusions [2]. A wide variety of organisms, including bacteria, viruses, prions, and parasites, can be transmitted through whole blood or blood product transfusions [3].

In low-income countries, TTIs are extremely common. Yet targeted screening for pathogens is limited in scope, serving only to address risk from a few, highly selected known pathogens. For example, the Ugandan Blood Transfusion Service (UBTS), similar to most other blood collection centers throughout sub-Saharan African, only screens blood for human immunodeficiency virus (HIV), hepatitis B virus (HBV), hepatitis C virus (HCV), and syphilis. Despite screening, retrospective testing has detected these viruses in screened blood due to inadequate methods and acute infections prior to marker positivity in blood [4]. Additional known viruses that may be transmitted by transfusion include human T-lymphotropic virus types I and II (HTLV-I and HTLV-II), hepatitis E virus (HEV), and arboviruses including West Nile virus, Zika virus, dengue virus, and human herpesvirus 8 (HHV-8) [5–11]. Furthermore, blood for transfusion throughout sub-Saharan Africa (SSA) is not screened for bacteria or parasites, despite regionally endemic infections that are known to be transfusion transmissible (e.g., malaria) [12]. Transfusion recipients remain susceptible to untested or unknown infectious agents. These limitations are amplified in regions of the world where blood safety testing may not be closely monitored or due to disruptions in laboratory supply chains such as during disease outbreaks (e.g., Ebola) or civil strife.

One innovative approach to reduce the threat posed by TTIs is the use of pathogen reduction technology (PRT). This technology has been approved by the European Medicines Agency (EMA), and a Conformitè Europëenne (CE) Mark was obtained for Mirasol-treated WB in 2015. In brief, Mirasol PRT, developed by Terumo BCT, requires riboflavin to be mixed into a whole blood (WB) unit, which is then exposed to UV light (313 nm) for 1 h [13]. The chemical reaction between riboflavin and UV light prevents DNA and RNA replication. Disruption of nucleic acids occurs through two independent mechanisms: (1) UV light breaking bonds in nucleic acids and (2) photo-activation of riboflavin molecules interacting with nucleic acids, leading to electron transfer and irreversible modifications to specific functional groups of nucleic acids (e.g., guanine) [14–16]. Agents with nucleic acids, including viruses, parasites, and bacteria, or white blood cells, are inactivated or destroyed by varying degrees. In vitro, Mirasol PRT reduces the infectious load in treated WB for most pathogens by 99.9% [14–18]. Data have demonstrated Mirasol reduces parasites (*Trypanosoma cruzi* [19], *Babesia microti* [20], *Babesia divergens, Leishmania donovani* [21], and *Plasmodium falciparum* [22]), bacteria (*Yersinia enterocolitica*, *Acinetobacter baumannii*, and *Serratia liquefaciens* [17, 23]), and viruses (HIV, HBV, and HCV [17, 18, 24, 25]) by three to seven logs. Destruction of WBCs reduces the risk of transfusion-associated graft-versus-host disease (TAGvHD) and is comparable to using gamma irradiation [26]. Cells without nucleic acids, i.e., RBCs and platelets, suffer only minor cellular damage while plasma proteins are well preserved [27, 28].

A single-center randomized trial of 226 participants in Ghana evaluated if Mirasol-treated WB reduces transfusion-transmitted malaria (TTM) [29]. The incidence of TTM was significantly lower in the Mirasol-treated WB group (1/28 [4%] compared to standard group 8/37 [22%]; *p* = 0.039). This small trial did not assess other TTIs or adverse outcomes that can only be evaluated with larger and longer trials. The objective of the Mirasol Evaluation of Reduction in Infections Trial (MERIT) is to investigate whether Mirasol PRT can prevent TTM as well as six other TTIs, namely HIV, HBV, HCV, HEV, HHV-8, and bacteria in vivo.

MERIT trial is a prospective, randomized, double-blind, controlled superiority clinical trial among individuals requiring WB transfusions in Uganda.

## Methods/design

Recruitment started in November 2019 and is expected to run until 2024. Two thousand patients will be enrolled and randomized (1:1) to receive either Mirasol-treated or standard-issue WB transfusions and followed up for 10 weeks. The clinical trial registration number is NCT03737669.

### Blood supply

The UBTS is the National Blood Service responsible for all blood transfusion and safety activities for the entire country. It is a semi-autonomous and centrally coordinated organization within the Ugandan Ministry of Health. It has a network of eight regional blood banks and six blood collection centers whose activities include donation, storage processing of blood, and distribution of processed blood to all health facilities whether government or private countrywide. Blood is distributed by UBTS to 277 health care facilities all over the country. In 2012, the UBTS collected 203,390 units of blood nationally. All blood donors (100%) are anonymous volunteers; there are no paid donors. UBTS tests all blood in a quality-controlled manner for HIV antigen-antibody, HBV surface antigen, HCV antibodies, and syphilis by rapid plasma reagin (RPR). TTI testing is done on blood that is drawn into the sample diversion pouch. Standard blood units/components supplied by UBTS are not leukoreduced nor are they gamma irradiated. In the standard arm of the trial, participants receive WB from regular collections that has undergone routine testing but has not been modified in any way. Intervention arm participants receive blood that in addition to the routine testing has been treated with Mirasol PRT illumination within 8 h of collection.

### Participants

The trial is being conducted by Makerere University-Johns Hopkins University Research Collaboration (MU-JHU) in Kampala, Uganda. The enrolment sites include the Mulago National Referral Hospital (MNRH), Kawempe Specialized National Referral Hospital (KSNRH), and Uganda Cancer Institute Mulago Specialised Women and Neonatal Hospital (UCI). The MNRH, located in Kampala, is the largest public referral and teaching hospital in Uganda with 1500 beds and approximately 3000 patients at any one time and includes the Orthopaedics Department, Surgery Department, and Casualty (i.e., Emergency) Unit. KSNRH is a public 170-bed hospital in Kampala that provides specialist obstetric, gynaecologic, and neonatal care. The UCI is the primary provider of specialized cancer treatment throughout Uganda.

Patients at these locations are eligible for the study if they meet the following inclusion criteria: (1) willing to participate in the study and the patient or legally authorized representative has given written informed consent; (2) transfusion necessary based on a hemoglobin of less than 7 g/dL or clinical judgment of attending physician following the national guidelines; (3) patient agrees to return to the hospital for follow-up visits; and (4) the doctor judges that the patient will be able to complete follow-up visits over the next 10 weeks.

Exclusion criteria (either permanent or temporary) are as follows: (1) presence of red cell alloantibodies; (2) incompatible red cell cross-match (at enrollment prior to first transfusion); (3) expected to require plasma or platelets within the next 10 weeks outside of the WB provided in the trial; (4) blood group Rhesus negative; (5) blood type AB (due to concern of limited supply); (6) weight < 30 kg (due to concern for sufficient blood draws to detect bacteria and other TTIs); (7) HIV-infected; (8) clinical suspicion of sepsis; (9) anti-malarial treatment or indication to be receiving malaria prophylaxis (e.g., sickle cell disease or ongoing pregnancy) within 7 days prior to randomization; (10) fever (central body temperature greater than 38.5 °C); (11) transfusion(s) of a blood product within 1 month prior to randomization; (12) acute or chronic medical disorder that, in the opinion of the investigator, would impair the ability of the patient to receive protocol treatment; and (13) previously enrolled and randomized in this trial or any other pathogen reduction trial.

### Randomization

Patients for whom a transfusion has been prescribed are referred to the study team by the attending physician or nurse. Participants who meet the eligibility criteria are assigned in a 1:1 ratio to either the control or treatment group using permuted block randomization. Participants may only be enrolled and randomized once. Randomization occurs only after confirming both Mirasol and control WB are available. The start of study treatment is defined as the initiation of the first WB transfusion. Participants receive all transfusions in the designated arm for up to 10 weeks after randomization.

### Blinding

Study personnel administering the transfusions and monitoring the participants for safety are blinded to the randomized treatment assignment. The study blood transfusion service laboratory technologists open the serial envelopes that have pre-assigned randomization to the study arm and study ID number. The technologists are responsible for assigning the participants the randomization and also for issuing the study blood. Mirasol-treated and standard WB differ slightly in the confirguation of the blood bag. Blinding of study staff and participants is maintained by use of blood bag cover. If it becomes necessary to unblind a specific participant’s assignment, e.g., for emergency medical management, the principal investigator will contact the technologists and obtain the treatment assignment of the subject. The Data and Safety Monitoring Board (DSMB) will be notified of the event within 2 business days.

### Assignment of intervention

The UBTS has a quality control program and follows the WHO recommended criteria for WB collection of 450 mL±10% with a hematocrit of 35–50% [30]. We are following all currently CE-marked, approved labeling criteria for standard WB or Mirasol-treated WB. Mirasol-treated units have an expiry date of 21 days assuming they are illuminated for PRT within 8 h of collection. For the trial, we also use standard units only up to 21 days and if not used, the units are returned to UBTS for re-issue as the expiry date for standard units is 35 days. Blood is stored at 1–6^o^C.

The decision to transfuse is made by the attending physician. Indications are specific to the individual patients and thus may vary by underlying pathology. The attending physician then refers the patient to the study. A study nurse/counselor gives the patient information about the study and obtains informed consent to join the study, and additional consent for sample storage and genetic testing is also sought. The participant is then screened for the study by a study doctor and samples drawn for testing. If found eligible, they are randomized and enrolled into the study.

**Fig. 1:**
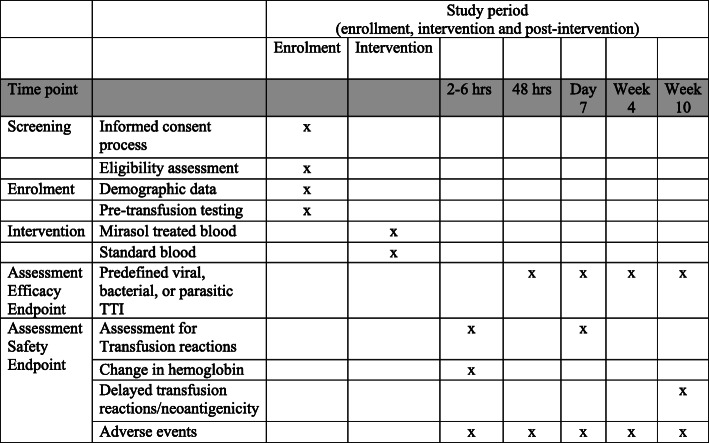
Schedule of enrolment, intervention, and assessments

**Fig. 2:**
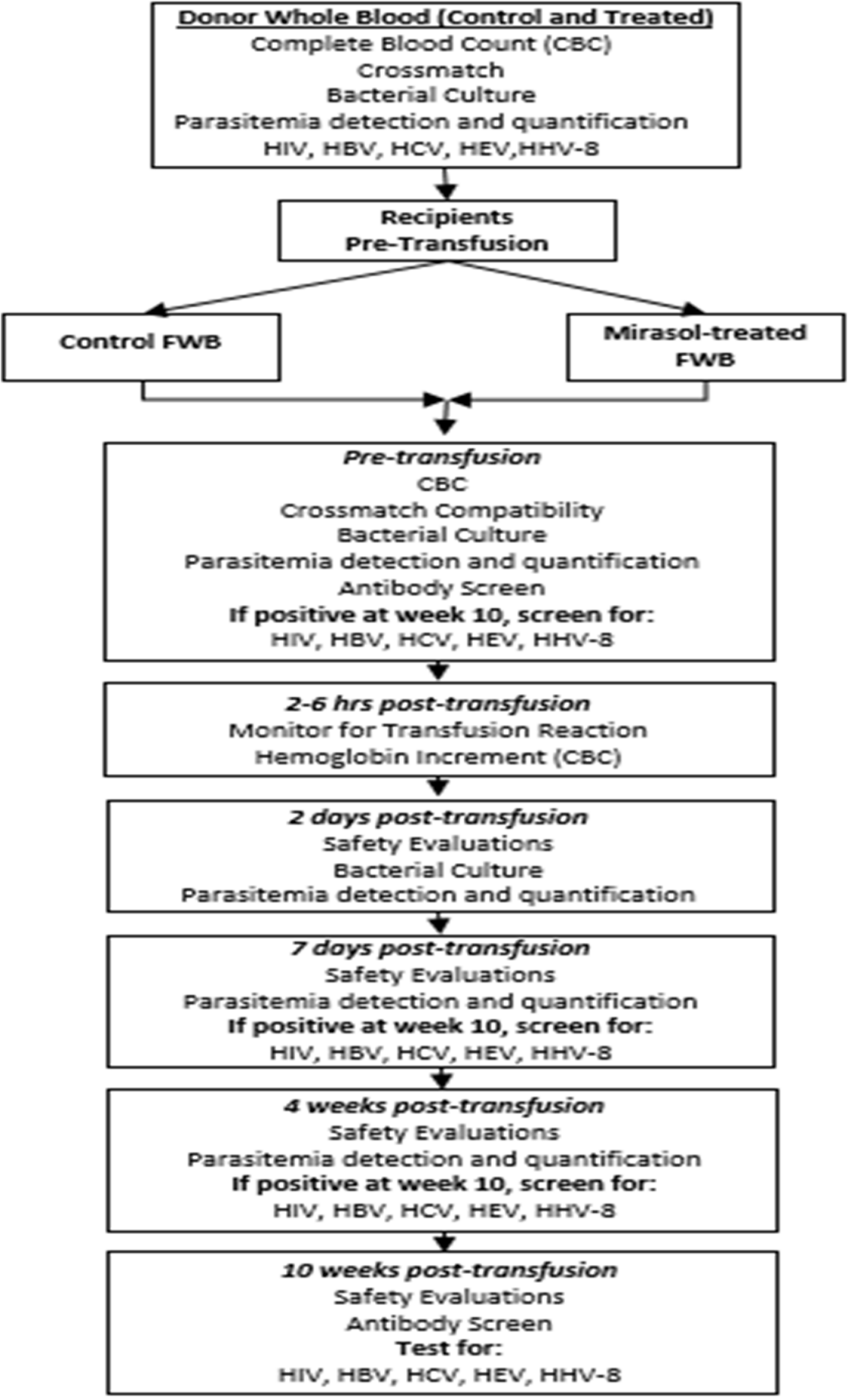
Donor blood and recipient evaluation

Transfusion recipients are followed up for 10 weeks and evaluated six times, five of which include the collection of blood samples (Figs. 1 and 2). The parentheses give window periods for sample draws:

1. *Pre-transfusion*: enrollment of the recipient and blood collected to evaluate baseline infectious status and RBC compatibility and antibody identification. RBC compatibility and antibody identification consist of verifying the ABO group of the unit, ABO/Rh testing and 37oC Coombs antibody screen for the recipient, and a serological cross-match at room temperature; in the event that a participant develops a clinically significant RBC alloantibody during the study and requires further transfusion, appropriate 37°C pre-transfusion testing/cross-match will be performed.
2. *At 2–6 h following transfusion*: assess hemoglobin increment with CBC and clinical evaluation for possible acute transfusion reactions, as defined by the US CDC Hemovigilance criteria [31].
3. *Within 48 h (+ 1 day)* of each transfusion: blood collected for bacterial culture and malaria identification.
4. *At 7 days (− 1 to + 2 days)* after the transfusion: obtain brief medical history focused on signs or symptoms of transfusion reactions and infections with HIV, HBV, HCV, HEV, HHV-8, or malaria. Blood collected for TTI testing and evaluation for transfusion reaction.
5. *At 4 weeks (−4 to + 7 days)* after the transfusion: obtain brief medical history focused on signs or symptoms of infection with HIV, HBV, HCV, HEV, HHV-8, or malaria. Blood collected for TTI testing.
6. *At 10 weeks (−7 to + 14 days)* after the first transfusion: obtain brief medical history focused on signs or symptoms of transfusion reactions and infections with HIV, HBV, HCV, HEV, HHV-8, or bacteria. Blood collected for TTI testing and evaluated for delayed transfusion reaction/new antibodies with antibody screen.

Individuals who require additional transfusions are followed as above as long as they receive the additional transfusions within the first 6 weeks of their initial transfusion (i.e., they will be evaluated at 48 h, day 7, and day 28 of both the first and any subsequent transfusions). The visits are combined if they fall within the same window. However, once the individual reaches week 10 from the initial enrollment and transfusion, their follow-up ends. If individuals require transfusion support for transfusions received after the first 6 weeks of study participation, they still receive study blood transfusions as per their randomization until study exit at 10 weeks but do not have additional follow-up visits. To encourage adherence to study procedures, participants are compensated for their time and transportation for each scheduled visit. Participants are also eligible to withdraw from the trial at any stage.

In case of a transfusion reaction, the transfusion is stopped and the patient evaluated. After the relevant checks and investigations have been done, the transfusion may or may not be restarted. The participant’s trial arm is maintained for all future transfusions. Participating in the trial should not affect the usual patient care or require any alteration to concomitant medication.

### Intervention outcomes

The primary endpoint of this study is the incidence of any pre-defined viral, bacterial, or parasitic TTI: HIV, HBV, HCV, HEV, HHV-8, bacteria, or malaria in a previously negative recipient for the specific TTI as indicated by changes in laboratory findings at day 2, day 7, week 4, or week 10 after the first transfusion. A TTI will only be ascribed when both the donor unit and recipient have the exact same pathogen as confirmed by specialized laboratory testing.

In addition, the clinical effectiveness of Mirasol-treated WB products compared to standard WB products in recipients will be evaluated by the following:

- Incidence and characteristics of acute transfusion reactions as defined by CDC Hemovigilance [31]
- Change in hemoglobin level post-transfusion compared to pre-transfusion
- Delayed transfusion reactions/neoantigenicity
  i) Incidence of an amnestic RBC alloantibody as detected by a positive direct antiglobulin test (DAT) when participants are evaluated for transfusion reaction
  ii) Incidence of new red cell alloantibodies post-transfusion (positive antibody screen at 10 weeks)
- Incidence of treatment-emergent adverse events (TEAE) and treatment-related adverse events

In order to assess the primary and secondary outcomes, the following laboratory tests will be performed (Fig. 1):

● Complete blood counts (CBCs)

● RBC antibody screen and direct antiglobulin test (DAT). The antibody screen will be used to determine new RBC alloantibody identification. If there is a suspected acute hemolytic transfusion reaction, a DAT will be performed. If the DAT is positive, antibody identification will be performed to determine whether it is a RBC alloantibody or a new antibody due to Mirasol PRT [32, 33].

● Bacteria: Cultures will be performed for both the donor unit and recipients. A TTI will only be ascribed when both the donor unit and recipient have the exact same genus and species.

● Viral TTIs: Serology assays for HIV (antigen and antibody combination), HBV (HBsAg, HBcAb), HCV (anti-HCV), HEV (IgG, IgM, and antigen), and HHV-8 (based on epitopes in open reading frames 73 and K8.1) will be performed on both donor units and recipients. The recipients will be evaluated at the last follow-up time point (week 10). If the recipient is positive, the other time points (i.e., enrollment (baseline), 48 h, day 7, and week 4) will subsequently be evaluated. HBV, HCV, HEV, and HIV nucleic acid testing (NAT) will also be performed among all donors and also for recipients at week 10, similar to serology testing.

● Malaria: Peripheral smears (thin and thick) will be performed among donor samples and on recipients at 48 h, day 7, and week 4. PCR assays will also be conducted to increase sensitivity [35]. The reading of the smears and PCR tests will be done retrospectively on stored samples and the results will not be available before transfusion. Two positive malaria PCR tests in the recipient post-transfusion samples are required to ascribe a malaria infection.

#### Analysis of HIV, HBV, HCV, and HEV by next-generation sequencing

For suspected transfusion transmission of HIV, HBV, HCV, and HEV, both donor and recipient samples will be analyzed by sequencing as previously described [34] to confirm viral phylogenetic linkage of a TTI between donor and recipient. Donor and recipient samples will be compared when both are confirmed positive by initial testing (i.e., serology or nucleic acid). The sequences will be compared to reference sequences, and aligned and phylogenetic trees will be created. A seroconversion event will be considered to be linked if all available index and partner samples contain consensus sequences in a monophylogenetic group with a high bootstrap value (> 70).

#### *Plasmodium* allelic discrimination

If parasitemic blood is transfused to a non-parasitemic patient and parasitemia is identified post-transfusion, representative genes of the *Plasmodium* genome will be amplified for comparison between the donor and recipient to determine if the parasitemia was transfusion-transmitted or community-acquired. Donor and recipient samples will be compared when both are confirmed positive by initial testing (i.e., peripheral smear or nucleic acid). Allelic discrimination will be conducted using nested PCR of informative genome regions to further identify *Plasmodium* species. Sequencing of the representative genes will further discriminate between *Plasmodium* present in transfused WB products and in the patient post-transfusion [36]. Further high-resolution analyses based on genomic methods will also be considered [37]. Transfusion transmission will be assessed by matching up to three alleles present in donor and patient. The polymorphism of each allele is variable and will be considered in the interpretation of transfusion-transmitted malaria.

### Study power calculations

Table 1 shows the prevalence of TTIs contributing to the primary endpoint in donated blood and the expected number of infections per 1000 transfused participants in Uganda. We anticipate that participants will receive 1.5 units of WB on average [43] with ~ 50% of participants receiving at least two transfusions. Given the low infectivity of some TTIs, we further assume the risk of TTIs is independent of one another. We anticipate ~ 50% of transfusion recipients will have prior immunity to HHV-8 and HEV; in the absence of prior information, we hypothesize (conservatively) that immunity to these viral infections is independent. Under the above conditions, the expected cumulative incidence of at least one TTI by 10 weeks is 3.7% among control subjects (31 infections among 850 participants after factoring in 15% loss to follow-up). More specifically, we estimate that the 31 infections in the control arm will be comprised of 13 malaria (42%), 10 bacteria (31%), and 8 viral TTIs (26%). Because of uncertainties over the prevalence of disease in the donor population, the rate of infectivity, and 10-week return rates (conservatively ~ 15% LTFU expected), we have designed the study to enroll up to 2000 participants with 1000 subjects per trial arm over a 30-month period. There will be an interim analysis after 1000 study subjects have been enrolled. See stopping rules below.

**Table 1:** Transfusion-transmitted infections in Uganda among blood donors. The table provides the prevalence of TTIs contributing to our primary endpoint in donated blood, sensitivity of screening methods (when used in Uganda), risk of a TTI given exposure to infected blood (i.e., infectivity), and the expected number of infections per 1000 transfused participants

Agent	Donor prevalence (%)	Screening method sensitivity (%)	Infectious units/1000 units of blood	Infectivity (%)	Expected number of infections/1000 transfused individuals+
HIV^#^	0.71	98	0.14	100	0.2
HBV^#^	2.41	98	0.48	75	0.5
HCV^#^	1.76	98	0.35	74	0.4
HEV*	0.6	No screening	6	75	6.8
HHV-8^	36.0	No screening	360	2.3	12.4
Malaria^∞^	5.0	No screening	50	20	15.0
Bacteria^≡^	1.5	No screening	15	50	11.3

^#^Seroprevalence data from Uganda Blood Transfusion Service in Kampala, Uganda, between April and June 2017

*HEV seroprevalence in Uganda is 47% [38]. However, RNA positivity with acute infection is ~ 0.6% [6]. Acute infection is most common among individuals in late adolescence, the primary blood donor age [39]

^HHV-8 seroprevalence in Uganda among late adolescence (primary blood donor age) is 36% [40]. The prevalence among recipients is ~ 50% [5]. There is an excess risk of HHV-8 seroconversion of 2.3% when transfused HHV-8 positive blood [5]

^∞^Malaria donor prevalence of 5% is a conservative estimate from data in Uganda [41]

^≡^Bacterial contamination prevalence derived from previous [42] and ongoing studies in Uganda of blood donors

+Expected number of infections in a population with no prior immunity

**Fig. 3:**
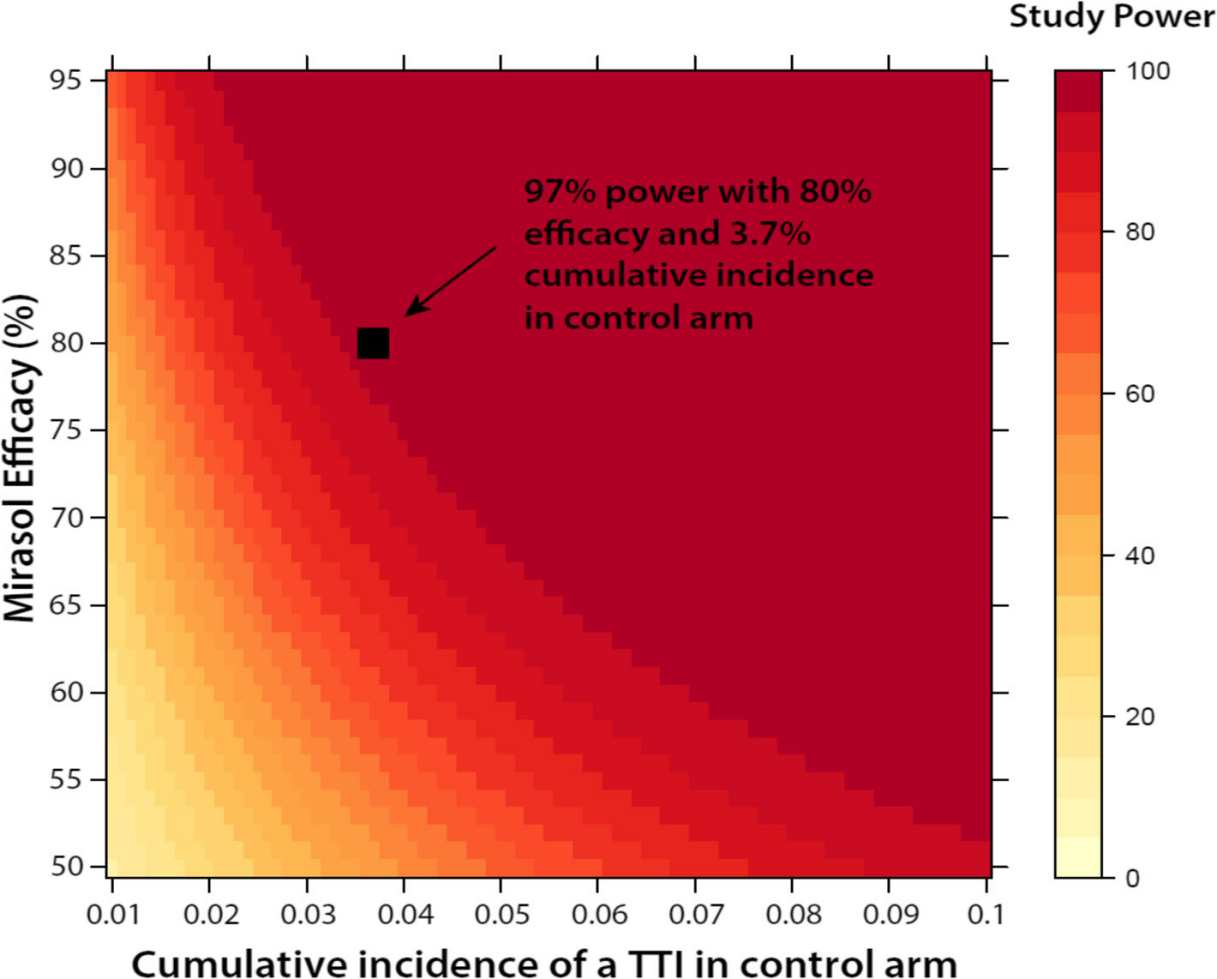
Study power based on Mirasol efficacy and cumulative TTI incidence

Figure 3 shows study power to detect a statistical difference in cumulative incidence of at least one TTI by 10 weeks between the intervention and control arms at varying levels of efficacy (*y*-axis; 50–90%) and cumulative incidence in the control arm (*x*-axis; 1–10%). Assuming a cumulative incidence of 3.7% in the control, an efficacy of 80% Mirasol PRT treatment (similar to the AIMS trial [29]), non-differential loss to follow-up of 15% by 10 weeks, we have 97% power to detect a statistically significant difference in cumulative TTI incidence between study arms (two-sided alpha = 0.05). Power to detect significant differences in cumulative incidence of malaria is 64%, viral infections 49%, and bacterial infections 51%.

### Data collection, entry, and storage

Data is collected both on hard copy (paper) case record forms and soft copy as e-forms, checked for errors and consistency, and entered into a Research Electronic Data Capture (REDCap) database. Each participant is assigned a screening and enrolment number which are used as identifiers on study documents, instead of names, for confidentiality purposes. Participants’ study files and other source documents are stored under lock and key. All data entered in the REDCap system is stored on a server using a password-protected system.

### Analysis plan

A modified intention to treat (mITT) analysis will be performed to determine whether Mirasol PRT treatment of WB decreases the incidence of TTIs. The primary analysis will evaluate recipients who become positive for at least one of the targeted TTIs during the follow-up period and who received a blood unit with the confirmed presence of the corresponding infectious agent. This will estimate what proportion of new infections can be avoided by adopting Mirasol in transfusion medicine. A secondary analysis will be performed evaluating the reduction of incident TTI that is genetically linked to the transfused unit, which will estimate the efficacy of Mirasol at reducing TTI incidence.

*The ITT population* will consist of all randomized participants who undergo at least one study transfusion, independent of the outcome or successful completion of the procedure. Summaries by treatment group will be based on the randomized treatment assignment. The modified ITT (mITT) population consists of all those in the ITT population who have been assessed and have results for at least one TTI.

The per protocol (PP) population is defined as those who:

1. Received WB transfusions per the randomized treatment allocation during the 10-week study period
2. Did not receive any non-study blood products during trial participation prior to confirmation of presence or absence of TTI
3. Did not receive any anti-malarial, anti-viral, or antibiotic treatment prior to confirmation of presence or absence of the malarial, viral, or bacterial TTI of interest, respectively
4. Have no significant protocol deviations (including, but not limited to, inclusion- and exclusion criteria, and missing critical blood samples for TTI testing)

The study is powered to evaluate the combined incidence of all TTIs assessed, but the impact of Mirasol PRT on malaria and viral and bacterial infections will also be assessed separately. We expect 31 cumulative infections in the control arm giving us sufficient power (> 90%) to detect differences between study arms for our primary endpoint. A minimum of 23 infections from both arms is required in order to ensure adequate study power (> 90% power to detect an efficacy of 80%).

*The primary endpoint* is the cumulative incidence of one or more TTIs by 10 weeks among the mITT population. Log-binomial regression models will be used to estimate the relative risk reduction of incident TTI by 10 weeks associated with the Mirasol PRT. If the log-binomial model fails to converge, a Poisson model with robust variance estimation will be specified. Individuals lost to follow-up (including those who miss one or more study visits) will be censored from the primary analysis. Sub-analyses will be conducted separately for adults and children (< 18 years) and by sex. The primary analysis will also be stratified by the number of transfusions, with participants who received 1–2 transfusions analyzed separately from those who received ≥3 transfusions. Stratified analyses will also be conducted by admitting hospital. Efficacy will also be analyzed at each follow-up visit.

Sensitivity analyses will be conducted to assess whether or not there were important differences in demographics or health status between those with and without complete follow-up. In the event there are differences between those lost to follow-up and those who completed all study visits, inverse probability censoring weighting and stratification will be used to assess potential selection biases [44]. Similar calculations will be made if there is differential follow-up. A sensitivity analysis will be performed among participants who completed their 10-week follow-up visit, regardless of any previously missed visits. An additional analysis will be performed restricted to participants who are negative for all bacterial, viral, and parasitic infections at baseline. A final sensitivity analysis will be performed among participants who attended at least one follow-up visit and test results for all TTI of interest are available. All analyses will be repeated in the ITT and PP populations.

#### Stopping rules

An interim analysis for efficacy with respect to the primary outcome (incidence of any study TTI) will be performed when 10-week outcome data are available for the first 1000 randomized transfusions. The Lan-DeMets implementation of the O’Brien-Fleming procedure will be used for this single interim analysis; the alpha level will be set to 0.00304, with the final testing alpha set at 0.04895 to maintain the overall alpha of 0.05 [45]. Under the protocol-specified efficacy of 80%, there is 49% power for this interim criterion to be met. If this criterion at 1000 is met, and at least 25% of the observed reduction is due to reduction in pathogens other than malaria, then stopping for efficacy may be recommended. A futility analysis will also be performed in the event the stopping rule is not met. Under the assumption that the future events are affected by the protocol-specified 80% reduction parameter, the conditional probability of the final *p*-value being less than 0.04895 will be calculated; if it is less than 10%, early termination may be recommended due to futility.

The Safety population will consist of all randomized subjects who undergo at least one study transfusion, independent of the outcome or successful completion of the procedure. Summaries by treatment group will be based on the treatment actually received. Specifically, the safety analyses will evaluate adverse transfusion reactions, delayed transfusion reactions, hemoglobin increments, treatment-related adverse events, and all-cause mortality. The trial might also be stopped if there are safety concerns.

#### Statistical methods for comparison of baseline study characteristics and safety outcomes

Continuous variables will be summarized with the mean, standard deviation, median and range, and 95% confidence intervals based on the normality assumption. Categorical variables will be summarized using proportions and exact (Clopper-Pearson) 95% confidence intervals will be provided for select summaries. The mITT population is the default population for the efficacy analyses and the Safety population is the default population for safety analyses. For categorical variables, statistical differences between the treatment arms will be assessed by chi-square test or Fisher exact test, as appropriate. For normally distributed continuous variables, Student’s *t*-test will be used to assess the statistically significant differences between the two treatment groups. For non-normal distributed data or where normalization is not possible, nonparametric tests will be used (e.g., Wilcoxon rank sum test), as appropriate. All statistical tests will be two-tailed.

Baseline and co-morbidity factors will be defined as the observations recorded prior to the first study transfusion and will be summarized for the Safety population by treatment received and according to the type of variable (categorical, continuous). Outcome parameters of safety and efficacy will be analyzed for the Safety and mITT populations, respectively, in a similar manner.

Baseline participant demographic and clinical characteristics will be summarized descriptively by study arm and overall. Baseline variables will include age, sex, weight, height, and BMI. The median number of transfusions per participant at the end of the follow-up will also be calculated. Summaries of these variables will be assessed among the mITT, ITT, and PP populations both overall and by treatment arm.

### Trial organization, monitoring, and safety

Quality and accuracy of the data are monitored and assured by FHI Clinical, an independent commercial Clinical Research Organization with extensive experience designing and managing clinical research in resource-constrained settings. Monitoring is conducted after every 100 new participants are enrolled.

The DSMB is comprised of individuals with expertise in transfusion medicine, infectious diseases, statistics, and clinical trial design and conduct. The DSMB sits regularly and reviews reports of trial data.

The severity of adverse experiences is graded using the NIH DAIDS Toxicity Guidelines version 2.0 [46]. All SAEs are reported in narrative form to the IRBs within 2 weeks of the site’s awareness of the event. Deaths are reported within 3 days of the site’s awareness, which is in line with the Uganda National Council of Science and Technology (UNCST) guidelines.

The study team will ensure the proper care and management of any adverse events related to the intervention arm.

### Dissemination

Participants will be informed about any positive results that could improve their own health, specifically treatable TTIs.

The research findings will be communicated through meetings with the study participants, conference presentations, and peer-reviewed publications to the scientific community and through reports and workshops to the funder and Ugandan Ministry of Health. Authorship will be determined via the International Committee of Medical Journal Editors (ICMJE) recommendations. Participant-level data will also be available from the corresponding authors.

## Discussion

Established TTIs, emerging, re-emerging, and novel pathogens all pose risk to global blood transfusion safety. This concern is heightened with the continued emergence of new pathogens worldwide. The pursuit of transfusion safety through targeted pathogen testing continues to add incremental cost to blood components without addressing the urgent need of emerging infections [47]. Additionally, even when screening tests are added for selected pathogens, patients remain at risk during the time taken for assessment, test development, and policy implementation. As one example, it took over a decade from recognition of risk to adoption of screening for *Babesia* in the USA [48, 49]. Pathogen reduction may be able to reduce some of these screening assays (e.g., syphilis) while responding to intense needs for blood supply.

Numerous factors have been cited for the accelerating threat of emerging infectious diseases (EID). These include the increase in access, ease, and speed of international travel; the effect of immigration on disease introduction in new regions; the inadvertent importation of disease vectors; the re-emergence of previously controlled infections; the increase in ranges for disease vectors due to climate change; the increased overlap between human and wild habitat; and the migration of animal hosts such as birds and deer [47, 50, 51]. Any infection with an asymptomatic blood-borne phase has the potential for transfusion transmission [50, 52]. Historically, the response to an EID has involved monitoring for disease emergence, identification of the disease-causing agent, analysis of epidemiological risk factors, definition of donor deferral criteria, and, if justified, development of a specific screening assay [50]. The current system for assuring the safety of the blood supply relies upon donor deferral procedures, EMA/FDA-licensed serological and/or nucleic acid testing assays that target specific transfusion-transmitted pathogens, and blood filtration procedures/leukoreduction. Not only is this inefficient, costly, and slow given the need for de novo test development or adaptation (replete with regulatory hurdles), it fails to address the complete spectrum of pathogens known, emerging, or re-emerging that threaten the blood supply. In contrast to the reactive strategy (deferral of donors, testing of donated blood) for blood safety, PRT offers a generalized, proactive mechanism to reduce or eliminate the threat of malaria and EID transmission by transfusion [47, 50]. The additional benefits of reducing alloimmunization, transfusion-associated graft versus host disease (TA-GvHD), and non-infectious transfusion reactions are also of high value.

Currently, no FDA-approved PRT system is available for use with WB or red blood cells, the two most commonly transfused blood products worldwide; the Mirasol PRT system is uniquely positioned to fill this gap. WB is used commonly in low-to-middle-income countries and in military trauma, but rarely in high-income countries; however, there is an increasing interest in its use for resuscitation in civilian trauma accompanied by massive hemorrhage because of comparable survival with when blood components are used and the logistical and economic advantages [43, 53–55]. Mirasol PRT has been shown to be effective across different classes of pathogens including bacteria, viruses, and parasites in vitro and for plasma and platelets [56]. If shown to be effective for WB, it could represent a fast, cost-effective way of making WB transfusions safe from both known and unknown pathogens.

There are potential limitations and challenges with MERIT. One potential limitation of this clinical trial may occur if the rate of TTIs in our study is lower than expected. However, we used conservative estimates for all of the TTIs. The study is also powered off the combined outcome of multiple TTIs, and the study power, at 97%, is robust to lower-than-expected incidence for any of these TTIs. In addition, there are risks of poor follow-up or participant loss due to mortality since many of the Ugandan patients requiring blood transfusion are very sick. However, MU-JHU closely engages study subjects and consistently has a < 10% loss with clinical trials requiring a 1–2-year follow-up. In addition, monitoring the blood inventory for both standard WB and Mirasol-treated WB is a challenge as the Mirasol-treated units only have a shelf life of 21 days (the same was adopted for the standard units to maintain blinding). Finally, the COVID-19 pandemic has affected all research globally and has not spared the MERIT trial; not only has it meant that the study staff are at risk of contracting the SARS-CoV2 virus while executing their duties, but the lock-downs and travel restrictions have meant scale down of activities at the recruitment sites and difficulties following participants that have to be overcome.

The MERIT trial will assess the efficacy of Mirasol PRT to reduce or eliminate TTIs from WB particularly in a resource-limited environment. If the trial demonstrates efficacy, PRT technology may represent a cost-effective, easy-to-use, and feasible technology whose implementation might improve the health of people especially in resource-limited settings or where a full blood screening program is not available.

## Trial status

The trial began recruitment at the beginning of the COVID-19 pandemic (November 2019), has recruited > 700 participants of the 2000 planned participants, and is expected to run till 2024. Since the statistical analysis plan was still being finalized, the protocol is just now being submitted.

## Data Availability

Consent forms and data will be available upon request from the principal investigator.

## Abbreviations

CBCs: Complete blood counts
CE: Conformitè Europëenne
DSMB: Data and Safety Monitoring Board
DAT: Direct antiglobulin test
EID: Emerging infectious diseases
EMA: European Medicines Agency
FDA: Food and Drug Administration
HBV: Hepatitis B virus
HCV: Hepatitis C virus
HEV: Hepatitis E virus
HHV-8: Human herpesvirus 8
HIV: Human immunodeficiency virus
HTLV-I and HTLV-II: Human T-lymphotropic virus types I and II
ITT: Intention to treat
ICMJE: International Committee of Medical Journal Editors
JCRCIRB: Joint Clinical Research Center Institutional Review Board
KSNRH: Kawempe Specialized National Referral Hospital
MU-JHU: Makerere University-Johns Hopkins University Research Collaboration
MERIT: Mirasol Evaluation of Reduction in Infections Trial
mITT: Modified ITT
MNRH: Mulago National Referral Hospital
NAT: Nucleic acid testing
PRT: Pathogen reduction technology
PP: Per protocol
RPR: Rapid plasma reagin
SSA: Sub-Saharan Africa
TAGvHD: Transfusion-associated graft-versus-host disease
TTIs: Transfusion-transmitted infections
TTM: Transfusion-transmitted malaria
UCI: Uganda Cancer Institute
UNCST: Uganda National Council of Science and Technology
UBTS: Ugandan Blood Transfusion Service
UNAIDS: United Nations Programme on HIV and AIDS
AMEDD: US Army Medical Department Board
WB: Whole blood
HRPO: US Department of Defense Human Research Protection Office
